# Clinical spectrum and treatment of thyroid lymphoma: results of a cohort study of 61 patients

**DOI:** 10.3389/fonc.2026.1720721

**Published:** 2026-06-10

**Authors:** Shuchang Li, Wenjie Gu, Zhenzhen Yang, Honghan Qiao, Feiyang Zong, Shifeng Hao, Yuxiao Chang, Wanyue Zhao, Xiang Gao, Xudong Zhang, Qingjiang Chen

**Affiliations:** 1 Department of Oncology, The First Affiliated Hospital of Zhengzhou University, Zhengzhou, China; 2 Henan Academy of Innovations in Medical Science, Zhengzhou, Henan, China

**Keywords:** chemotherapy, prognostic factors, surgery, thyroid lymphoma, thyroid neoplasm

## Abstract

**Background:**

Thyroid lymphoma (TL) is a rare malignancy. Its diagnosis is challenging due to non-specific clinical features, with management controversies, concurrent with Hashimoto’s thyroiditis (HT), and heterogeneous prognoses across subtypes. This study aimed to analyze clinical spectrum and explore optimal treatment strategies to improve prognosis of TL.

**Methods:**

A retrospective cohort study was conducted on 61 patients with TL diagnosed in the First Affiliated Hospital of Zhengzhou University from 2014 to 2025.

**Results:**

Among 61 patients, 67.2% had concurrent HT. The TL+ group (TL with extrathyroid involvement, 11 patients, 18.0%) showed more frequent subdiaphragmatic lymph node involvement (p<0.001) and perivascular lymph node involvement (p=0.003) compared with the iTL group (isolated TL, 50 patients, 82.0%). With a median follow-up of 22 months, the 1-, 3-, and 5-year OS rates were 85.0%, 78.4%, and 75.6%, and PFS rates were 76.5%, 71.7%, and 63.1%. DLBCL was the most common histological subtype, accounting for 60.7% of all TL cases. In patients with thyroid diffuse large B-cell lymphoma (TDLBCL), age ≥60 years and C-myc expression ≥50% were independent risk factors for shorter PFS, whereas chemotherapy was independently associated with improved PFS and OS. Surgery alone without chemotherapy was associated with shorter PFS in univariate analysis.

**Conclusions:**

TL with extrathyroidal involvement shares features with isolated disease but requires Ann Arbor staging-guided management. Chemotherapy was independently associated with improved PFS and OS in patients with thyroid diffuse large B-cell lymphoma, whereas older age and high C-myc expression were independently associated with inferior PFS.

## 1 Introduction

Thyroid lymphoma (TL) is a rare thyroid malignancy, accounting for approximately 1% to 5% of all thyroid cancers and less than 2% of all extranodal lymphomas (1). TL is staged using the Ann Arbor system: Stage IE is defined as lymphoma limited to the confines of the thyroid gland, stage IIE represents spread beyond the thyroid to regional lymph nodes, stage IIIE represents involvement of lymph nodes on both sides of the diaphragm, and stage IVE indicates systemic dissemination (2). Primary thyroid lymphoma (PTL) is conventionally defined as lymphoma confined to the thyroid or the thyroid with adjacent cervical lymph nodes, without contiguous spread or distant metastasis from other areas at diagnosis (3). The application of modern imaging techniques, such as PET-CT (positron emission tomography-computed tomography), has facilitated staging while introducing new complexities. When multiple extranodal organs are involved at initial diagnosis, identifying the primary lesion can be challenging, because extranodal lymphomas in different anatomical sites may differ in cellular origin, pathogenesis, clinical presentation, treatment strategy, and prognosis (4). Regions with higher SUVmax(Standardized Uptake Value Maximum) typically indicate the highest tumor activity, serving as preferred sites for biopsy sampling (5). Given the clinical features of PTL, challenges in diagnosis, disparities in treatment strategies and prognosis across stages, most studies have focused on limited-stage PTL (stages I–II). For stage I–II PTL, localized disease extent enables favorable treatment responses, and early diagnosis allows timely intervention to prevent progression to advanced stages, thereby significantly improving the long-term survival. Additionally, exploring lower-intensity or more localized therapies in early stages may reduce treatment-related toxicities and complications, enhancing patients’ quality of life (6). Considering the difficulty in identifying the primary lesion in lymphomas involving multiple extranodal organs (including the thyroid), our study did not emphasize the distinction of primary thyroid lymphoma. Instead, we included all patients with lymphoma confirmed by thyroid tissue biopsy at first visit. Based on imaging evidence of the involvement of other extranodal organs, we stratified TL into two groups: cases with extrathyroid involvement at diagnosis (TL+) and cases without other extranodal organ involvement (iTL). Clinical characteristics, treatment and survival outcomes were analyzed and compared between two groups.

Optimal treatment strategies for TL remain controversial because of the paucity of randomized and prospective trials guiding clinical practice. Given the diagnostic challenges, frequent comorbidities, and complexity in selecting treatment strategies, comprehensive management of TL necessitates multidisciplinary collaboration involving surgery, endocrinology, oncology, and hematology. This study analyzed TL patients at our center to characterize the clinical features, explore treatment modalities and identify prognostic factors for thyroid diffuse large B-cell lymphoma (TDLBCL). This study aimed to explore the clinical features and prognostic factors of TL, in order to provide new references for basic research and clinical diagnosis and treatment of the rare extranodal malignant lymphoma.

## 2 Materials and methods

### 2.1 General data

We performed a retrospective analysis which was on clinical data of patients with pathologically confirmed thyroid lymphoma at the First Affiliated Hospital of Zhengzhou University from October 2014 to June 2025. Thyroid lymphoma was diagnosed based on histopathology and immunohistochemistry of thyroid tissue, in accordance with the 2022 WHO classification of lymphoid neoplasms. Patients with incomplete data, loss to follow-up after discharge, or concurrent other malignancies were excluded. A total of 61 patients were included in this study.

Collected data included the following: age at first visit, gender, initial symptoms, Eastern Cooperative Oncology Group (ECOG) performance status, The Ann Arbor stage, International Prognostic Index (IPI) for non-Hodgkin lymphoma, pathological subtype, Hans classification, immunohistochemical positivity rates of Ki67, BCL-2, and C-myc, serum levels of lactate dehydrogenase (LDH) and β2-microglobulin, thyroid antibody levels, thyroid endocrine function, ultrasound features of intrathyroidal tumors, and treatment modalities.

TL+ was defined as thyroid lymphoma with involvement of other extranodal organs at diagnosis, whereas iTL was defined as thyroid lymphoma without other extranodal organ involvement, regardless of nodal involvement.

The definitions of treatment modalities used in this study are as follows: Chemotherapy exposure was defined as receipt of at least one cycle of systemic chemotherapy or immunochemotherapy. Completion of standardized chemotherapy was defined as completion of at least four cycles of a standard first-line systemic regimen, including R-CHOP, CHOP, R-EPOCH, EPOCH, BR, R-DA-EPOCH, or other approved first-line protocols according to histological subtype and clinical condition. Surgery was defined as any surgical procedure involving resection of the thyroid lesion, performed for diagnostic purposes, relief of airway compression, or as part of multimodal management. Radiotherapy was defined as local radiation therapy directed to the thyroid gland and/or cervical or mediastinal lymph node regions, administered as adjuvant, consolidation, or palliative treatment. Multimodal therapy was defined as the combination of two or more treatment modalities, including chemotherapy, surgery, and radiotherapy.

### 2.2 Follow-up

Follow-up data were obtained by reviewing medical records, imaging, and laboratory results from multiple previous hospitalizations and outpatient visits via the electronic medical record system of the First Affiliated Hospital of Zhengzhou University, supplemented by telephone interviews. The follow-up period started from the date of pathological confirmation and ended on June 30, 2025.

This study was approved by the Medical Ethics Committee of the First Affiliated Hospital of Zhengzhou University (Approval No. 2022-KY-0869-001). Owing to the retrospective nature of the study and the use of anonymized patient data, the requirement for written informed consent was waived in accordance with relevant national legislation and institutional regulations. This study was conducted in accordance with the Declaration of Helsinki.

### 2.3 Statistical analysis

Overall survival (OS) was measured from the date of diagnosis until death of any cause or the last follow-up. Progression-free survival (PFS) was measured from the date of diagnosis until the date of disease progression, relapse, or death of any cause. Response assessment was defined per the Lugano classification: complete response (CR), partial response (PR), stable disease (SD), and progressive disease (PD). ORR was defined as the proportion of patients achieving CR or PR. All survival analyses were performed using the Kaplan-Meier method and compared using the log-rank test. Pearson’s χ² test or Fisher’s exact test was used to assess differences in categorical variables. Univariate and multivariate analyses were performed using the Cox proportional hazards regression model. Statistical analysis was performed using SPSS 27.0 and GraphPad Prism 10.1.2. A *p* value of <0.05 was considered statistically significant.

## 3 Results

### 3.1 Baseline characteristics of thyroid lymphoma

A total of 61 patients with TL were enrolled in this study, including 50 cases in the iTL group and 11 cases in the TL+ group. Baseline characteristics of the overall cohort are summarized in **Table 1**.

**Table 1:** Baseline characteristics of patients with thyroid lymphoma (n=61).

Characteristic		Total (n=61)	iTL (n=50)	TL+ (n=11)	*χ²*-value	*p-value*
Demographics	Male	19 (31.1%)	14 (28.0%)	5 (45.5%)	1.281	0.258
	Age, median (range), years	61 (31–87)	62.5 (31–87)	53 (32–80)	–	–
	≥60 years	32 (52.5%)	28 (56.0%)	4 (36.4%)	–	0.323*
Clinical Features	IPI >2	20 (32.8%)	15 (30.0%)	5 (45.5%)	0.977	0.323
	ECOG >1	11 (18.0%)	9 (18.0%)	2 (18.2%)	–	1.000*
	Ann Arbor stage III–IV	17 (27.9%)	6 (12.0%)	11 (100.0%)	–	<0.001*
	Involvement					
	Extranodal sites ≥2	11 (18.0%)	0 (0.0%)	11 (100.0%)	–	<0.001*
	Subdiaphragmatic lymph nodes	13 (21.7%)	4 (8.0%)	9 (81.8%)	–	<0.001*
	Perivascular lymph nodes	5 (8.2%)	1 (2.0%)	4 (36.4%)	–	0.003*
	Bone marrow	5 (8.2%)	0 (0.0%)	5 (45.5%)	–	<0.001*
	Ki-67 ≥70%	33 (54.1%)	27 (54.0%)	6 (54.5%)	0.001	0.974
	Hashimoto’s thyroiditis	41 (67.2%)	35 (70.0%)	6 (54.5%)	0.977	0.323
Symptoms	Asymptomatic at diagnosis	30 (49.2%)	24 (48.0%)	6 (54.5%)	0.155	0.694
	B symptoms	2 (3.3%)	1 (2.0%)	1 (9.1%)	–	0.331*
	Significant compression	22 (36.1%)	18 (36.0%)	4 (36.4%)	–	1.000*
	Tracheal	7 (11.5%)	6 (12.0%)	1 (9.1%)	–	1.000*
	Esophageal	8 (13.1%)	5 (10.0%)	3 (27.3%)	–	0.148*
	Nerve involvement	7 (11.5%)	6 (12.0%)	1 (9.1%)	–	1.000*
	Pain	7 (11.5%)	6 (12.0%)	1 (9.1%)	–	1.000*
Laboratory Results	LDH elevated	26 (42.6%)	20 (40.0%)	6 (54.5%)	0.780	0.377
	β2-microglobulin elevated	9 (14.8%)	7 (14.0%)	2 (18.2%)	–	0.659*
	Thyroid dysfunction	32 (52.5%)	24 (48.0%)	8 (72.7%)	–	0.188*
	TPOAb positive	33 (54.1%)	29 (58.0%)	4 (36.4%)	–	0.317*
	TgAb positive	39 (63.9%)	33 (66.0%)	6 (54.5%)	0.513	0.474
Imaging Features	Tumor diameter, median (range), mm	47.5 (6–109)	46.3 (6–109)	52.0 (10–88)	–	–
	≥75 mm	15 (24.6%)	11 (22.0%)	4 (36.4%)	–	0.439*
	Multifocal lesions	41 (67.2%)	34 (68.0%)	7 (63.6%)	–	1.000*
	Bilateral involvement	40 (65.6%)	33 (66.0%)	7 (63.6%)	–	1.000*
	Diffuse	6 (9.8%)	3 (6.0%)	3 (27.3%)	–	0.066*

Values are presented as n (%) for categorical variables. The χ² test or Fisher’s exact test was used for group comparisons. “*” indicates Fisher’s exact test was used. “-” indicates no available data. Continuous variables are presented as median (range) and were not included in group comparison when no p-value is shown.Abbreviations: IPI, International Prognostic Index; ECOG, Eastern Cooperative Oncology Group; LDH, lactate dehydrogenase; TPOAb, thyroid peroxidase antibody; TgAb, thyroglobulin antibody.

TL was more common in females (68.9%), with a predominance of middle-aged individuals. The median age was 61 years (range, 31–87 years), and 52.5% of patients were ≥60 years. No statistically significant differences were observed in terms of gender distribution or the proportion of patients aged ≥60 years between the iTL and the TL+ group. DLBCL (diffuse large B-cell lymphoma) was the most common pathological subtype (37 patients, 60.7%), followed by 11 with MALT (mucosa-associated lymphoid tissue) lymphoma, 3 with FL (follicular lymphoma), and 2 with Burkitt lymphoma, with different distributions between the two groups. Notably, all indolent TL cases were in the iTL group. Patients with Ki-67≥70%, IPI>2 and ECOG performance status>1 accounted for 54.1%, 32.8% and 18.0%, respectively. Subdiaphragmatic lymph node involvement (p < 0.001) and perivascular lymph node involvement (p=0.003) were more frequent in the TL+ group than in the iTL group, supporting the more invasive phenotype of TL +.

For diagnosis, 30 patients (49.2%) were asymptomatic, presenting only with thyroid nodules or goiter detected on physical examination, while the remaining 31 cases had conditions such as local compression (22 patients, 36.1%), pain (7 patients, 11.5%), and B symptoms (2 patients, 3.3%). All patients underwent thyroid function testing and antibody assays: 41 (67.2%) had concurrent Hashimoto’s thyroiditis; 32 (52.5%) had abnormal thyroid function; 33 (54.1%) were positive for thyroid peroxidase antibody (TPOAb); and 39 (63.9%) were positive for thyroglobulin antibody (TgAb). On ultrasonography, the median tumor diameter was 47.5 mm. No statistically significant differences were observed between the two groups in terms of tumor size≥75 mm, multifocality, or bilateral involvement. Only the difference in patients with diffuse ultrasonographic features between the two groups approached statistical significance (p=0.066), with a higher proportion in the TL+ group (3 cases [6.0%] in iTL vs. 3 [27.3%] in TL+). Thus, among baseline characteristics, significant differences between the two groups were limited to subdiaphragmatic and perivascular lymph node involvement, consistent with the tendency of TL+ to involve multiple extranodal sites.

### 3.2 Diagnosis, treatment, and prognosis of the iTL group and TL+ group

At the first visit, 22 patients (36.0%) underwent fine needle aspiration (FNA), and only 4 cases were definitively diagnosed via FNA. The remaining patients underwent core needle biopsy or surgical resection for adequate tissue sampling and histological confirmation. Details of treatment modalities are shown in **Table 2**. Most patients received chemotherapy (51 patients, 83.6%). Forty-six patients (75.4%) completed standardized chemotherapy with at least four cycles of first-line systemic therapy. R-CHOP was the most commonly used regimen in the overall cohort, administered to 30 patients (49.2%), including 23 patients (46.0%) in the iTL group and 7 patients (63.6%) in the TL+ group (p=0.335). Other regimens included CHOP in 3 patients (4.9%), R-EPOCH in 2 patients (3.3%), EPOCH in 1 patient (1.6%), BR in 4 patients (6.6%), R-DA-EPOCH in 1 patient (1.6%) and other therapies. Treatment response was evaluable in 51 patients and is summarized in **Table 3**. The ORR was numerically higher in the iTL group than in the TL+ group, although the difference did not reach statistical significance (92.7% vs. 70.0%, Fisher’s exact test, p=0.081). Among the 10 patients who did not receive chemotherapy, 8 underwent surgery alone, 1 received radiotherapy, and 1 received lenalidomide alone due to hepatitis B. Additionally, 5 patients discontinued chemotherapy due to adverse events.

**Table 2:** Diagnosis, treatment, survival outcomes of thyroid lymphoma (n=61).

Characteristic	total (n=61)	iTL (n=50)	TL+ (n=11)	*χ²*-value	*p-value*
Diagnosis					
DLBCL	37 (60.7%)	30 (60.0%)	7 (63.6%)	–	–
Hans GCB subtype	22 (36.1%)	18 (36.0%)	4 (36.4%)	–	–
HGBL	5 (8.2%)	3 (6.0%)	2 (18.2%)	–	–
MALT lymphoma	11 (18.0%)	11 (22.0%)	0 (0.0%)	–	–
FL	3 (4.9%)	3 (6.0%)	0 (0.0%)	–	–
Burkitt lymphoma	2 (3.3%)	2 (4.0%)	0 (0.0%)	–	–
Other	3 (4.9%)	1 (2.0%)	2 (18.2%)	–	–
Biopsy methods					
FNA	22 (36.0%)	19 (38.0%)	3 (27.3%)	–	0.731*
CNB	34 (55.7%)	28 (56.0%)	6 (54.5%)	0.008	0.930
Resection	29 (47.5%)	24 (48.0%)	5 (45.5%)	0.023	0.878
Treatment					
Chemotherapy exposure	51 (83.6%)	41 (82.0%)	10 (90.9%)	–	0.673*
Completed standardized chemotherapy	46 (75.4%)	36 (72.0%)	10 (90.9%)	–	0.265*
First-line chemotherapy regimen					
R-CHOP	30 (49.2%)	23 (46.0%)	7 (63.6%)	-	0.335*
CHOP	3 (4.9%)	3 (6.0%)	0 (0.0%)	-	-
R-EPOCH	2 (3.3%)	2 (4.0%)	0 (0.0%)	-	-
EPOCH	1 (1.6%)	1 (2.0%)	0 (0.0%)	-	-
BR	4 (6.6%)	4 (8.0%)	0 (0.0%)	-	-
R-DA-EPOCH	1 (1.6%)	1 (2.0%)	0 (0.0%)	-	-
Others	10 (16.4%)	7 (14.0%)	3 (27.3%)	-	-
Surgery	29 (47.5%)	24 (48.0%)	5 (45.5%)	0.023	0.878
Surgery only (no chemo)	10 (16.4%)	9 (18.0%)	1 (9.1%)	–	0.673*
Radiotherapy	11 (18.0%)	8 (16.0%)	3 (27.3%)	–	0.400*
Radiotherapy only	1 (1.6%)	1 (2.0%)	0 (0.0%)	–	–
CNS prophylaxis	3 (4.9%)	1 (2.0%)	2 (18.2%)	–	0.081*
Multi-modal therapy	21 (34.4%)	16 (32.0%)	5 (45.5%)	0.723	0.395
Survival Outcomes					
Progression	21 (34.4%)	16 (32.0%)	5 (45.5%)	0.723	0.395
Death	15 (24.6%)	12 (24.0%)	3 (27.3%)	–	1.000*

Values are presented as n (%) for categorical variables. The χ² test or Fisher’s exact test was used for group comparisons. “*” indicates Fisher’s exact test was used. “-” indicates not applicable or not calculated.Abbreviations: TL+, extrathyroid involvement group; iTL, no other extranodal organ involvement group; DLBCL, diffuse large B-cell lymphoma; MALT, mucosa-associated lymphoid tissue; FL, follicular lymphoma; FNA, fine needle aspiration; CNB, core needle biopsy; CNS, central nervous system.

**Table 3:** Treatment response in evaluable patients with thyroid lymphoma according to extranodal organ involvement (n=51).

Response	Total (n=51)	iTL (n=41)	TL+ (n=10)	*p-value*
ORR	45 (88.2%)	38 (92.7%)	7 (70.0%)	0.081*
CR	30 (58.8%)	27 (65.9%)	3 (30.0%)	
PR	15 (29.4%)	11 (26.8%)	4 (40.0%)	
SD	1 (2.0%)	0 (0.0%)	1 (10.0%)	
PD	5 (9.8%)	3 (7.3%)	2 (20.0%)	

Values are presented as n (%). Percentages were calculated using the number of evaluable patients in each column as the denominator. Treatment response was assessed at the end of first-line treatment according to the Lugano classification. For patients who discontinued first-line treatment prematurely or changed regimens, the last available radiological assessment before discontinuation or regimen change was used to determine treatment response. ORR was defined as CR plus PR. “*” indicates Fisher’s exact test.Abbreviations: TL, thyroid lymphoma; iTL, thyroid lymphoma without other extranodal organ involvement; TL+, thyroid lymphoma with other extranodal organ involvement; ORR, objective response rate; CR, complete response; PR, partial response; SD, stable disease; PD, progressive disease.

**Figure 1:**
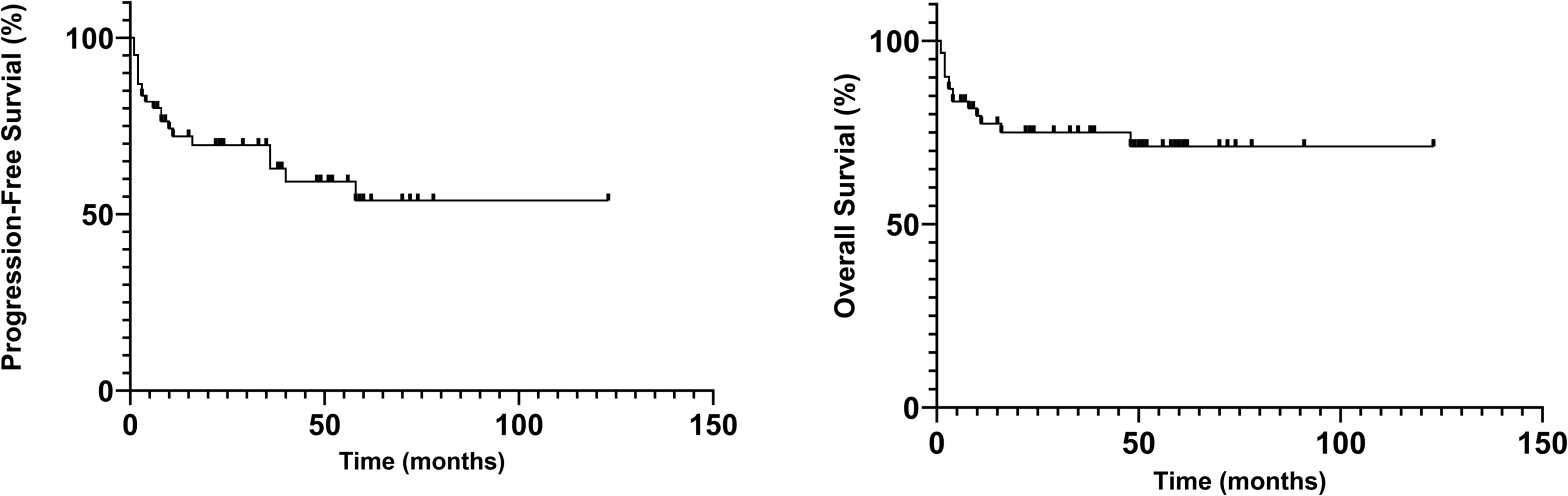
Progression-free survival and overall survival of the total patient group.

**Figure 2:**
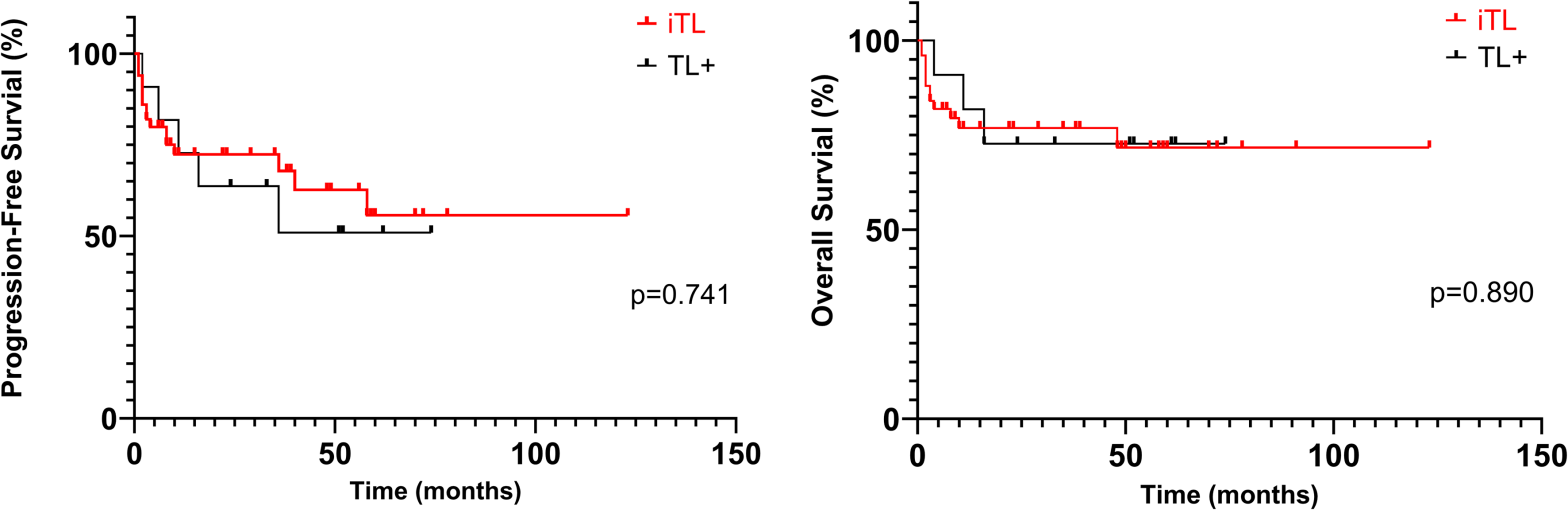
Progression-free survival and overall survival of the extrathyroid involvement group(TL+) and no other extranodal organ involvement group (iTL).

With a median follow-up of 22 months, the 1-, 3-, and 5-year OS rates were 85.0%, 78.4%, and 75.6%, respectively; the corresponding PFS rates were 76.5%, 71.7%, and 63.1% (**Figure 1**). Among the 46 patients (75.4%) who received standard chemotherapy regimens, 41 (67.2% of the entire cohort) received rituximab-containing regimens, with a median of 6 cycles for first-line treatment. Of the 29 patients who underwent surgery, 10 (16.4%) received surgery alone without chemotherapy. During follow-up, 7 of these 10 developed disease progression: 4 died, 1 survivor had not undergone treatment after progression, while the remaining 2 received chemotherapy after recurrence and achieved remission. The remaining 3 patients remained stable. Eleven patients (18.0%) received radiotherapy, among whom 1 patient with severe tracheal stenosis succumbed. In terms of treatment modalities, no significant differences between the iTL and TL+ groups were observed. Only 1 TL+ patient developed central nervous system (CNS) progression. However, among the 3 patients who underwent CNS prophylaxis, 2 were in the TL+ group (p=0.081), suggesting a potential trend toward differential use of this treatment, which requires confirmation in larger cohorts. Notably, none of the 3 patients (4.9%) who received CNS prophylaxis developed CNS progression. No significant difference in survival outcomes was observed between the iTL and TL+ groups(**Figure 2**).

### 3.3 Prognostic factors of diffuse large B-cell lymphoma of the thyroid

Among the 37 patients with TDLBCL, R-CHOP remained the predominant first-line regimen, used in 22 patients (59.4%), followed by CHOP in 3 patients (8.1%), R-EPOCH in 2 patients (5.4%), EPOCH in 1 patient (2.7%), and other regimens in 4 patients (10.8%); 5 patients (13.5%) did not receive chemotherapy (**Table 4**). Treatment responses according to first-line chemotherapy regimens are shown in **Table 5**. Among chemotherapy-treated patients with TDLBCL, the ORR was 95.5% in the R-CHOP group and 70.0% in the non-R-CHOP chemotherapy group. Although R-CHOP was associated with a numerically higher ORR, the difference was not statistically significant (Fisher’s exact test, p=0.079). Given the small number of patients treated with each non-R-CHOP regimen, response rates were summarized descriptively and no further regimen-specific comparisons were performed.

**Table 4:** Treatment regimens and treatment modalities in patients with thyroid diffuse large B-cell lymphoma (n=37).

Treatment category	Regimen or approach	Total (n=37)	iTL (n=30)	TL+ (n=7)	p-value
First-line therapy	R-CHOP	22 (59.5%)	17 (56.7%)	5 (71.4%)	0.677*
	CHOP	3 (8.1%)	3 (10.0%)	0 (0.0%)	–
	R-EPOCH	2 (5.4%)	2 (6.7%)	0 (0.0%)	–
	EPOCH	1 (2.7%)	1 (3.3%)	0 (0.0%)	–
	Other regimens	4 (10.8%)	3 (10.0%)	1 (14.3%)	–
Treatment modality	Chemotherapy	32 (86.5%)	26 (86.7%)	6 (85.7%)	1.000*
	Rituximab-containing first-line regimen	28 (75.7%)	22 (73.3%)	6 (85.7%)	0.656*
	Radiotherapy	8 (21.6%)	6 (20.0%)	2 (28.6%)	0.631*
	Surgical resection	14 (37.8%)	11 (36.7%)	3 (42.9%)	1.000*
Maintenance therapy	Any maintenance therapy	12 (32.4%)	9 (30.0%)	3 (42.9%)	0.659*
	Rituximab maintenance	9 (24.3%)	8 (26.7%)	1 (14.3%)	0.656*
	Lenalidomide maintenance	5 (13.5%)	3 (10.0%)	2 (28.6%)	0.233*
	No maintenance therapy	25 (67.6%)	21 (70.0%)	4 (57.1%)	0.659*

Values are presented as n (%). Percentages were calculated using the number of patients in each column as the denominator. Maintenance therapy categories were not mutually exclusive because some patients received both rituximab and lenalidomide maintenance. “Any maintenance therapy” was defined as receipt of rituximab and/or lenalidomide maintenance. “No maintenance therapy” was defined as receipt of neither rituximab nor lenalidomide maintenance. “*” indicates Fisher’s exact test.Abbreviations: TDLBCL, thyroid diffuse large B-cell lymphoma; iTL, thyroid lymphoma without other extranodal organ involvement; TL+, thyroid lymphoma with other extranodal organ involvement; R-CHOP, rituximab, cyclophosphamide, doxorubicin, vincristine, and prednisone; CHOP, cyclophosphamide, doxorubicin, vincristine, and prednisone; R-EPOCH, rituximab, etoposide, prednisone, vincristine, cyclophosphamide, and doxorubicin.

**Table 5:** Treatment response according to first-line chemotherapy regimen in patients with TDLBCL receiving chemotherapy (n=32).

Group	n	CR	PR	SD	PD	ORR
R-CHOP	22	14 (63.6%)	7 (31.8%)	0 (0.0%)	1 (4.5%)	21 (95.5%)
Non-R-CHOP chemotherapy	10	3 (30.0%)	4 (40.0%)	0 (0.0%)	3 (30.0%)	7 (70.0%)

Values are presented as n (%). Percentages were calculated using the number of patients in each treatment group as the denominator. Response was assessed according to the Lugano classification. ORR was defined as CR plus PR. Non-R-CHOP chemotherapy included CHOP, R-EPOCH, EPOCH, and other non-R-CHOP systemic regimens. The ORR was numerically higher in the R-CHOP group than in the non-R-CHOP chemotherapy group, although the difference did not reach statistical significance (95.5% vs. 70.0%, Fisher’s exact test, p=0.079).

**Figure 3:**
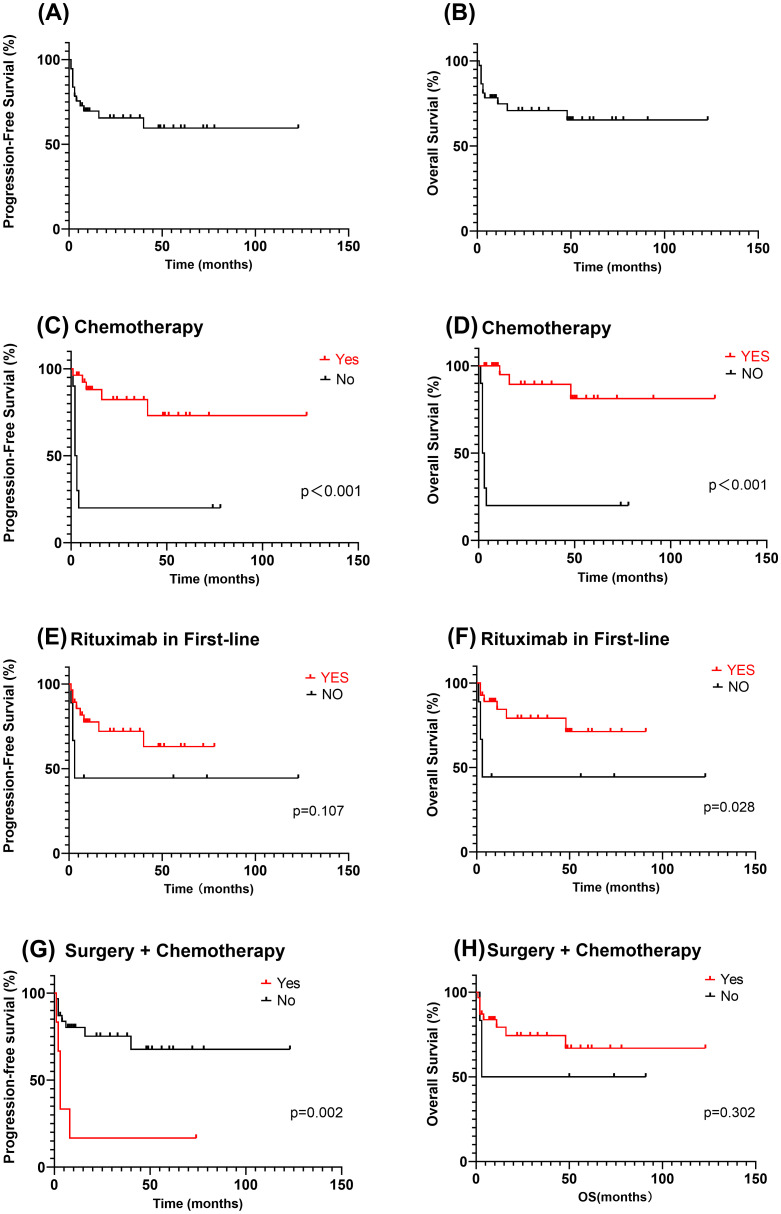
**(A, B)** Univariate PFS and OS curves in thyroid diffuse large B-cell lymphoma; **(C, D)** PFS and OS stratified by whether patients received first-line systemic chemotherapy (Yes vs No); **(E, F)** PFS and OS stratified by first-line rituximab-containing immunotherapy (Yes vs No); **(G, H)** PFS and OS stratified by combined surgery plus chemotherapy treatment versus non-combined management (Yes vs No). Log-rank test P values are presented within each corresponding panel.

With a median follow-up of 22 months, 13 PFS events and 11 OS events occurred. Because of the limited number of patients receiving each non-R-CHOP regimen, survival analysis according to individual chemotherapy protocols was not statistically robust. Therefore, treatment-related prognostic analyses focused on chemotherapy administration, rituximab in first-line, surgery alone, and multimodal therapy. We stratified patients with DLBCL into subgroups based on different treatment modalities. Kaplan–Meier survival curves according to treatment subgroups are shown in **Figure 3**.

**Figure 4:**
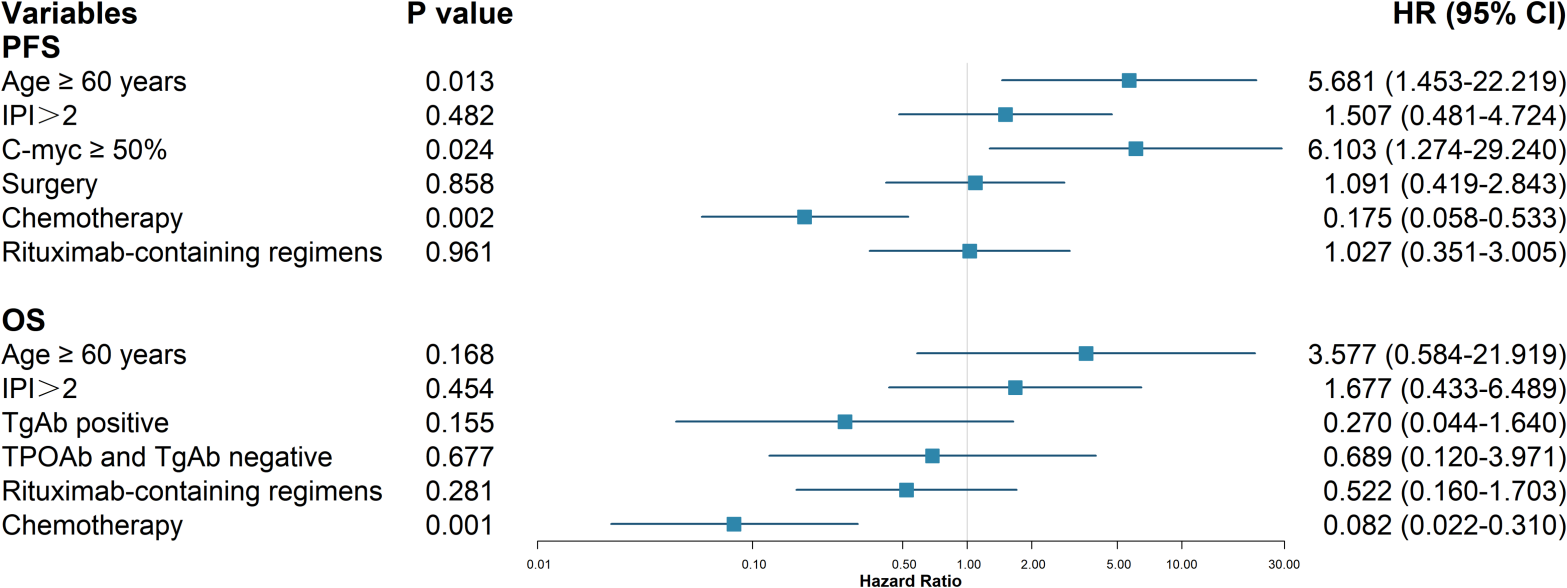
Forest plot of multivariate Cox regression analyses for prognostic factors in patients with thyroid diffuse large B-cell lymphoma. The plot displays hazard ratios (HR) and their 95% confidence intervals (CI) for factors associated with progression-free survival (PFS) and overall survival (OS). Each square represents the HR estimate, and the horizontal line represents the 95% CI. The vertical line at HR = 1 indicates no effect. The forest plot was generated using R software (version 4.5.2).Abbreviations: IPI, International Prognostic Index; TgAb, thyroglobulin antibody; TPOAb, thyroid peroxidase antibody.

The detailed results of univariate and multivariate analyses are presented in **Table 6** and **Figure 4**, respectively.

**Table 6:** Univariate analysis of prognostic factors in patients with thyroid diffuse large B-cell lymphoma (n=37).

Factor category	Specific factor	PFS		OS	
		χ² value	*P* value	χ² value	*P* value
Demographics	Male	2.394	0.122	1.062	0.303
	Age ≥ 60 years	10.051	0.002	8.322	0.004
Pathology	Hans GCB	0.088	0.767	0.166	0.684
	Ki - 67 ≥ 70%	0.281	0.596	0.311	0.577
	BCL2 ≥ 40%	3.243	0.072	2.983	0.084
	C-myc ≥ 50%	4.048	0.044	1.441	0.230
	BCL2 + C-myc co-expression	1.646	0.199	0.768	0.381
Clinical features	ECOG >1	0.201	0.654	0.855	0.355
	Ann Arbor Stage III - IV	0.134	0.715	0.071	0.790
	IPI>2	6.847	0.009	11.260	0.001
	Hashimoto’s thyroiditis	1.060	0.303	2.582	0.108
	Asymptomatic	0.199	0.655	0.101	0.750
	Significant compression	0.004	0.947	0.097	0.756
Invasion sites	Non - regional lymph nodes	0.005	0.944	0.112	0.738
	Bone marrow	0.021	0.885	0.194	0.660
	Extranodal organs ≥ 2	0.109	0.741	0.019	0.890
Laboratory tests	TPOAb positive	0.116	0.734	0.933	0.334
	TgAb positive	2.014	0.156	4.034	0.045
	TPOAb and TgAb both positive	0.693	0.405	1.282	0.258
	TPOAb and TgAb both negative	0.995	0.318	4.309	0.038
	LDH elevated	0.649	0.421	2.905	0.088
	β2-microglobulin elevated	0.005	0.943	0.028	0.868
	Thyroid dysfunction	1.378	0.240	3.043	0.081
Ultrasound features	Diffuse	0.313	0.576	0.496	0.481
	Bilateral	0.052	0.819	0.583	0.445
	Multifocal lesions	0.054	0.816	0.494	0.482
	Tumor diameter ≥ 75 mm	0.585	0.444	0.022	0.883
Treatment	Surgery alone	10.213	0.001	2.219	0.136
	Chemotherapy alone	1.804	0.179	0.148	0.700
	Multimodal therapy	3.657	0.056	3.785	0.052
	Rituximab-containing first-line regimens	5.651	0.017	7.798	0.005
	Radiotherapy	1.564	0.211	0.330	0.565
	Surgery	0.352	0.553	0.291	0.590
	Chemotherapy	19.280	< 0.001	29.759	< 0.001
	Maintenance with Rituximab	3.622	0.057	3.548	0.060
	Maintenance with lenalidomide	1.509	0.219	1.674	0.196

Survival differences were assessed using the Kaplan–Meier method and compared using the log-rank test. χ² values represent log-rank χ² statistics. P values <0.05 were considered statistically significant.Abbreviations: OS, overall survival; PFS, progression-free survival; GCB, germinal center B-cell; IPI, International Prognostic Index; LDH, lactate dehydrogenase; TPOAb, thyroid peroxidase antibody; TgAb, thyroglobulin antibody.

Univariate survival analysis identified several factors associated with survival outcomes in patients with TDLBCL. Factors significantly associated with PFS included age ≥60 years (χ²=10.051, p=0.002), C-myc expression ≥50% (χ²=4.048, p=0.044), IPI >2 (χ²=6.847, p=0.009), surgery alone without chemotherapy (χ²=10.213, p=0.001), rituximab-containing regimens (χ²=5.651, p=0.017), and chemotherapy administration (χ²=19.280, p<0.001).

For OS, significant factors included age ≥60 years (χ²=8.322, p=0.004), IPI >2 (χ²=11.260, p=0.001), TgAb positivity (χ²=4.034, p=0.045), negative TPOAb/TgAb status (χ²=4.309, p=0.038), rituximab-containing regimens (χ²=7.798, p=0.005), and chemotherapy administration (χ²=29.759, p<0.001). Other variables, including Hashimoto’s thyroiditis, Ann Arbor stage III–IV, LDH, BCL2 expression ≥40%, radiotherapy, surgical resection, and maintenance therapy, were not significantly associated with survival outcomes.

In the multivariate Cox regression model, age ≥60 years (HR = 5.681, 95% CI 1.453–22.219, p=0.013) and C-myc expression ≥50% (HR = 6.103, 95% CI 1.274–29.240, p=0.024) were independent risk factors for shorter PFS, whereas chemotherapy was independently associated with improved PFS (HR = 0.175, 95% CI 0.058–0.533, p=0.002). For OS, chemotherapy remained the only independent protective factor (HR = 0.082, 95% CI 0.022–0.310, p=0.001). Other variables, including IPI >2, surgery, rituximab-containing regimens, TgAb positivity, and TPOAb/TgAb double-negative status, did not retain independent significance.

## 4 Discussion

TL is a rare and highly heterogeneous malignancy, with its clinical behavior, treatment strategies, and prognosis predominantly dictated by histological subtype and disease stage.

Normal thyroid tissue lacks lymphoid tissue, yet up to 80% of TL patients have concurrent HT. Patients with HT have a 40 to 80-fold increased risk of developing TL compared with the general population, with TL typically arising 20–30 years after the initial diagnosis of HT. Notably, although approximately 80% of PTL patients have concurrent HT, only 0.6% of individuals with HT progress to PTL (7–9). It is postulated that this association arises from the development of intrathyroidal lymphoid tissue in HT. This acquired lymphoid tissue, which resembles MALT, may evolve into non-Hodgkin lymphoma (10). Controversy exists regarding the impact of HT on TL prognosis. Both Belal et al. and Onal et al. reported improved outcomes in HT-associated TL. Belal et al. demonstrated a higher recurrence-free survival rate in patients with pre-existing thyroiditis (94% vs. 47% without thyroiditis). Onal et al. observed superior disease-free survival (DFS), OS, and local control (LC) in such patients, attributing this to the association of thyroiditis with MALT lymphoma or MALT-transformed DLBCL—subtypes with more indolent behavior than other DLBCLs (11, 12). In our cohort, 67.2% of TL patients had concurrent HT, with a higher proportion observed among patients with TDLBCL (70.3%). Although the incidence of HT did not differ significantly across TL subtypes (p=0.344), HT was not significantly associated with either PFS or OS in patients with TDLBCL. These findings suggest that HT may be more relevant to the pathogenesis and diagnostic background of TL than to survival stratification in this small retrospective cohort. Recently, Demircioğlu et al. reported a case of primary thyroid diffuse large B-cell lymphoma with clinical and histopathological features consistent with the diagnostic challenges discussed in the present study (13). TgAb positivity and negative TPOAb/TgAb status were associated with OS in univariate analysis, but neither remained independently associated with OS in multivariate analysis. We propose that HT may be a critical risk factor for TL, likely via inducing chronic thyroid inflammation and lymphocytic infiltration—creating a permissive microenvironment for lymphoma development in the lymphoid tissue–naive thyroid (7).

TL typically presents as a rapidly enlarging mass, often with compressive symptoms such as dysphagia, dyspnea, or hoarseness, while only a minority exhibit lymphoma-related B symptoms (fever, night sweats, weight loss). Ultrasonographically, TL manifests as a hypoechoic, ill-defined mass with heterogeneous texture, but these features are non-specific, overlapping with HT (diffuse enlargement with hypoechogenicity) and thyroid carcinoma (ill-defined borders, compressive symptoms) (14). Histopathological confirmation is the gold standard for TL. FNA serves as a preliminary diagnostic method but has limited sensitivity due to potential confusion between lymphoma cells, thyroiditis infiltrates, and other malignancies. This diagnostic challenge is more prominent in TL patients with concurrent HT, as the chronic inflammatory background of HT may mask the typical features of TL, increasing the risk of misdiagnosis. Meanwhile, the cellular atypia of some highly aggressive TL subtypes may be misidentified as anaplastic thyroid carcinoma, leading to misdiagnosis. This underscores that for patients with rapidly enlarging thyroid masses or compressive symptoms, if routine examinations fail to confirm the diagnosis, clinicians should perform FNA or even core needle biopsy(CNB) to obtain histological evidence, so as to avoid misdiagnosis or delayed treatment due to non-specific symptoms (15, 16). In our study, Of the 22 patients underwent FNA, only 4(18.2%) achieved a definitive diagnosis, highlighting this limitation. FNA is a screening tool, not a substitute for CNB or surgical biopsy—methods providing adequate tissue for histological and immunohistochemical analyses, which enabled definitive diagnosis in most TL cases in our cohort. Although 36% of patients were initially diagnosed with lymphoproliferative disorders via FNA, most required further tissue sampling for definitive diagnosis, consistent with the limitations of FNA when used alone. Notably, combining FNA with flow cytometry, cell block preparation for immunohistochemistry, and fluorescence *in situ* hybridization (FISH) for gene rearrangement detection may reduce reliance on surgical biopsy for diagnosing DLBCL and shorten the time from clinical presentation to treatment initiation; Su MW et al. reported a median of 51 days vs. 46 days with needle aspiration (15, 17).

TL with involvement of other extranodal organs showed clinical features broadly comparable to TL without other extranodal organ involvement, although it was associated with more frequent subdiaphragmatic and perivascular lymph node involvement. While early-stage disease may be amenable to localized therapy, advanced-stage cases typically demand intensified multimodal strategies to mitigate the risk of distant recurrence (18). In our cohort, patients in the iTL and TL+ groups exhibited similar clinical characteristics, with no prognostic differences across subgroups (p=0.395). Notably, the distinction between iTL and TL+ in this study relied on diagnostic imaging, but due to the retrospective nature of the study, not all patients underwent PET/CT(38, 62.3%). Even so, in patients diagnosed with TL involving extrathyroidal sites, PET/CT showed that the thyroid was the region with the highest SUVmax, suggesting the thyroid gland as the dominant lesion.

The British Thyroid Association guidelines state that the overall prognosis of thyroid lymphoma is “generally excellent”; however, prognosis is subtype-dependent, with 5-year survival rates as low as 45% (19). MALT lymphoma generally confers excellent long-term outcomes, with 5-year survival rates exceeding 90%, whereas DLBCL prognosis is influenced by treatment intensity, molecular markers (BCL2/BCL6 expression), and response to frontline therapy (12).

With the advent of multi-modal adjuvant therapy and growing focus on targeted therapy research, the management and prognosis of PTL have evolved. Standard chemotherapy for non-Hodgkin lymphoma involves the anthracycline-based CHOP (cyclophosphamide, doxorubicin, vincristine, prednisone) regimen combined with rituximab. Data from our center showed that 47.5% of TL patients underwent surgery before first-line treatment. Among patients with TDLBCL who underwent surgical resection, those managed with surgery alone without subsequent chemotherapy had significantly shorter PFS in univariate analysis (p=0.001), whereas OS was not significantly different (p=0.136). Of 6 chemotherapy-naive patients, 1 remained stable, 2 achieved stability after post-recurrence chemotherapy, and 3 died of rapid progression. Although the small sample size limited statistical power, the observed survival pattern suggests that surgery alone may be insufficient for patients with TDLBCL who are eligible for systemic therapy. During follow-up, 46 (75.4%) TL patients received at least first-line treatment. PFS and OS did not differ between 14 DLBCL patients who underwent surgery plus chemotherapy vs. 14 who received chemotherapy alone, indicating no additional benefit from combined surgery and chemotherapy. Eleven (18.0%) patients received radiotherapy: 9 for persistent disease, 1 died of complications after radiotherapy for airway stenosis, and 1 achieved complete remission with first-line radiotherapy. Other studies report divergent outcomes: Rosen et al. (61 cases) noted better local control (80% vs. 40%) and lower distant recurrence (7% vs. 23%) with residual lesions <2.5 cm. Belal et al. recommended 3–4 cycles of CHOP followed by consolidation radiotherapy (30–36 Gy) for stage IE/IIE disease. Yufan Tang et al. (propensity score-matched cohort) found comparable disease-specific survival (DSS) between surgery alone and combination therapy in stage IE PTL, with fewer treatment-related complications, shorter treatment duration, and lower costs—suggesting that adjuvant radiotherapy and/or chemotherapy may be unnecessary after surgery for stage IE PTL (7, 15).

In this cohort study of 61 TL patients, we characterized the clinical heterogeneity of this rare malignancy, with a focus on outcomes in iTL versus TL+, and identified key prognostic factors in DLBCL, the most common subtype. Patients with TL involving other extranodal organs at diagnosis but presenting predominantly with thyroid-related manifestations may require management principles similar to those used for PTL, while treatment intensity should still be guided by Ann Arbor stage, histological subtype, performance status, and multidisciplinary assessment. In patients with TDLBCL, age ≥60 years and IPI >2 were associated with both PFS and OS in univariate analysis, whereas C-myc expression ≥50% was associated with shorter PFS. Treatment-related factors were strongly associated with outcomes: chemotherapy and rituximab-containing regimens were associated with improved PFS and OS in univariate analysis, whereas surgery alone without chemotherapy was associated with inferior PFS. In multivariate analysis, age ≥60 years and C-myc expression ≥50% remained independent adverse prognostic factors for PFS, while chemotherapy was independently associated with improved PFS and OS. We acknowledge the role of surgery in TL patients, particularly in diagnosis and airway management; however, patients with DLBCL underwent surgery without subsequent chemotherapy had significantly shorter PFS but not OS. In terms of lymphoma prognosis and survival, TL patients should be managed by individualized, multidisciplinary protocols. Our study has certain limitations: due to its retrospective design, inherent selection bias, and the rarity of TL, the generalizability of our findings may be limited. Future studies with larger cohorts and molecular profiling are needed to refine prognostic models and develop management strategies for this heterogeneous disease.

## 5 Conclusion

TL with extrathyroidal involvement shares features with isolated disease but requires Ann Arbor staging-guided management: early-stage disease may respond to localized therapy, while advanced stages need intensified multimodal strategies. In thyroid DLBCL, surgery remains important for diagnosis and airway management, but surgery alone appears insufficient for patients eligible for systemic therapy. Chemotherapy was independently associated with improved PFS and OS, whereas age ≥60 years and C-myc expression ≥50% were independently associated with inferior PFS.

## Data Availability

The original contributions presented in the study are included in the article/supplementary material. Further inquiries can be directed to the corresponding authors.
